# Supplementary material for: Germline determinants of humoral immune response to HPV-16 protect against oropharyngeal cancer
Source: Nat Commun. 2021 Oct 12;12:5945. doi: 10.1038/s41467-021-26151-9 (PMC8511029; doi:10.1038/s41467-021-26151-9)

Supplementary Table 1: Description of epidemiological studies in the analysis.

Study Name	Location	PI	Study Design	PMID	Controls, N	OPC, N	OCC, N
Carolina Head and Neck Cancer Epidemiology (Chance)	USA (North Carolina)	A.F.Olshan	Population-based case-control	20049634	581	215	268
Alcohol-Related Cancers and Genetic susceptibility in Europe (ARCAGE)	Croatia, Czech Republic, Germany, Greece, Ireland, Italy, Norway, Spain, UK	P. Brennan	Multicenter Hospital-based case-control	18830131	729	196	413
IARC Central Europe	Poland, Romania, Russia, Slovakia	P. Brennan	Multicenter Hospital-based case-control	16614111	100	0	61
European Prospective Investigation into Cancer and Nutrition (EPIC)	France, Germany, Greece, Italy, Netherlands, Spain, Sweden, UK	M. Johansson, P. Brennan	Multicenter cohort study	12639222	1570	0	56
IARC oral cancer (ORC)	Canada, Italy, Poland, Spain	R. Herrero	Multicenter Hospital-based case-control	14652239	175	0	100
Maastricht	Netherlands	M. Lacko, W. Peters	Hospital-based case-control	21351260	274	0	103
University of Pittsburgh	USA (Pittsburgh)	B. Diergaarde	Hospital-based case-control	23619143	782	242	454
Rome	Italy	S. Boccia	Hospital-based case-control	20861397	93	0	56
Toronto	Canada	R. Hung G. Liu	Hospital-based case-control	24519853	952	456	403
Head and neck 5000 (HN5000)	UK	A. Ness	Multicenter clinical cohort	25519023	0	534	445
Overall					5256	1643	2359

Supplementary Table 2: OPC HPV16 E6 and L1 seropositive seroprevalences by study

Study Name	OPC			
	E6(+)/ Total	E6%	L1(+)/ Total	L1%
Carolina Head and Neck Cancer Epidemiology (Chance)	159/215	74.0	131/215	60.9
Alcohol-Related Cancers and Genetic susceptibility in Europe (ARCAGE)	65/196	33.2	33/195	16.9
University of Pittsburgh	185/242	76.4	148/242	61.2
Toronto	315/456	69.1	251/456	55.0
Head and neck 5000 (HN5000)	354/534	66.3	204/534	38.2
Overall	1078/1643	65.6	767/1642	46.7

Supplementary Table 3. Demographic characteristics for: HPV(+) versus HPV(-) OPC stratified by study.

ARCAGE			HPV(-)OPC		HPV(+)OPC	
		All n (%)	n	%	n	%
Total		196 (100)	131	66.8	65	33.2
Gender	Females	48 (24.5)	33	25.2	15	23.1
	males	148 (75.5)	98	74.8	50	76.9
Age	≤50	39 (19.9)	29	22.1	10	15.4
	50-59	86 (43.9)	56	42.7	30	46.2
	60-69	54 (27.6)	36	27.5	18	27.7
	≥70	17 (8.7)	10	7.6	7	10.8
	NA	-	-	-	-	-
	Median (IQR)	57.0 (52.0-64.0)	57.0 (51.0-63.0)		58.0 (53.0-65.0)	
Smoking status	Never	25 (12.8)	8	6.1	17	26.2
	Former	45 (23.0)	23	17.6	22	33.8
	Current	126 (64.3)	100	76.3	26	40.0
	NA	-	-	-	-	-
Drinking status	Never	9 (4.6)	5	3.8	4	6.2
	Ever	187 (95.4)	126	96.2	61	93.8
	NA	-	-	-	-	-

CHANCE			HPV(-)OPC		HPV(+)OPC	
		All n (%)	n	%	n	%
Total		215	56	26.0	159	74.0
Gender	Females	34 (15.8)	12	21.4	22	13.8
	males	181 (84.2)	44	78.6	137	86.2
Age	≤50	64 (29.8)	13	23.2	51	32.1
	50-59	93 (43.4)	22	39.3	71	44.7
	60-69	42 (19.5)	17	30.4	25	15.7
	≥70	16 (7.4)	4	7.1	12	7.5
	NA	-	-	-	-	-
	Median (IQR)	55.0 (49.5-61.0)	58.5 (51.75-65.0)		54.0 (48.0-60.0)	
Smoking status	Never	49 (22.8)	4	7.1	45	28.3
	Former	88 (40.9)	21	37.5	67	42.1
	Current	78 (36.3)	31	55.4	47	29.6
	NA					
Drinking status	Never	22 (10.2)	3	5.4	19	11.9
	Ever	193 (89.8)	53	94.6	140	88.1
	NA	-	-	-	-	-

Pittsburgh			HPV(-)OPC		HPV(+)OPC	
		All n (%)	n	%	n	%
Total		242 (100)	57	23.6	185	76.4
Gender	Females	46 (19.0)	19	33.3	27	14.6
	males	196 (81.0)	38	66.7	158	85.4
Age	≤50	60 (24.8)	11	19.3	49	26.5
	50-59	95 (39.3)	19	33.3	76	41.1
	60-69	69 (28.5)	18	31.6	51	27.6
	≥70	18 (7.4)	9	15.8	9	4.9
	NA	-	-	-	-	-
	Median (IQR)	56.9 (51.0-62.5)	59.9 (52.7-65.1)		56.1 (55.6-61.7)	
Smoking status	Never	82 (33.9)	10	17.5	72	38.9
	Former	76 (31.4)	12	21.1	64	34.6
	Current	84 (34.7)	35	61.4	49	26.5
	NA	-	-	-	-	-
Drinking status	Never	42 (17.4)	6	10.5	36	19.5
	Ever	200 (82.6)	51	89.5	149	80.5
	NA	-	-	-	-	-

Toronto			HPV(-)OPC		HPV(+)OPC	
		All n (%)	n	%	n	%
Total		456 (100)	141	30.9	315	69.1
Gender	Females	72 (15.8)	31	22.0	41	13.0
	males	384 (84.2)	110	78.0	274	87.0
Age	≤50	77 (16.9)	13	9.2	64	20.3

	50-59	172 (37.7)	53	37.6	119	37.8
	60-69	147 (32.2)	49	34.8	98	31.1
	≥70	60 (13.2)	26	18.4	34	10.8
	NA	-	-	-	-	-
	Median (IQR)	58.8 (53.2-65.6)	60.7 (55.0-67.2)		58.1 (52.4-64.6)	
Smoking status	Never	119 (26.1)	12	8.5	107	34.0
	Former	216 (53.9)	66	46.8	150	47.6
	Current	121 (27.2)	63	44.7	58	18.4
	NA	-	-	-	-	-
Drinking status	Never	67 (14.7)	14	9.9	53	16.8
	Ever	362 (79.4)	115	81.6	247	78.4
	NA	27 (5.9)	12	8.5	15	4.8

HN5000			HPV(-)OPC		HPV(+)OPC	
		All n (%)	n	%	n	%
Total		534 (100)	180	33.7	354	66.3
Gender	Females	112 (21.0)	38	21.1	74	20.9
	males	424 (79.0)	142	78.9	280	79.1
Age	≤50	97 (18.2)	26	14.4	71	20.1
	50-59	197 (36.9)	57	31.7	140	39.5
	60-69	174 (32.6)	66	36.7	108	30.5
	≥70	65 (12.2)	31	17.2	34	9.6
	NA	1 (0.2)	0	0	1	0.3
	Median (IQR)	58.9 (52.9-65.1)	61.4 (54.8-66.5)		58.1 (52.5-63.8)	
Smoking status	Never	116 (21.7)	20	11.1	96	27.1
	Former	215 (40.3)	60	33.3	155	43.8
	Current	70 (13.1)	47	26.1	23	6.5
	NA	133 (24.9)	53	29.4	80	22.6
Drinking status	Never	109 (20.4)	34	18.9	75	21.2
	Ever	291 (54.5)	90	50.0	201	55.8
	NA	134 (25.1)	56	31.1	78	22.0

Supplementary Table 4: Background characteristics of the oral cavity participating subjects after quality control.

	Oral cavity cancer cases no. (%)	Control no. (%)	p-value*
Oncoarray individuals passed QC	2359	5256	
Geographic region			0.003
Europe	1224 (51.89)	2927 (55.69)	
North America	1135 (48.11)	2329 (44.31)	
Age			0.33
≤50	442 (18.74)	889 (16.91)	
51-60	674 (28.57)	1561 (29.7)	
61-70	722 (30.61)	1649 (31.37)	
>70	521 (22.09)	1157 (22.01)	
Unknown	0 (-)	0 (-)	
Sex			0.08
Male	1546 (65.54)	3334 (63.43)	
Female	813 (34.46)	1922 (36.57)	
Unknown	0 (-)	0 (-)	
Smoking status			<0.001
Never	490 (22.83)	2019 (40.9)	
Former	701 (32.67)	1821 (36.89)	
Current	955 (44.5)	1096 (22.2)	
Unknown	213 (-)	320 (-)	
Drinking status			0.51
Never	410 (18.48)	846 (17.83)	
Ever	1809 (81.52)	3898 (82.17)	
Unknown	140 (-)	512 (-)	

OC cases definition: C02.0–C02.9 (except C02.4), C03.0–C03.9, C04.0–C04.9, C05.0–C06.9

* Logistic regression models were implemented to test associations of each variable.

Supplementary Table 5: Descriptive parameters for HPV antibody assays in cases and controls.

HPV Serology Marker	Cutoff (MFI)	OPC MFI values median IQR	Controls MFI values median IQR
HPV6L1	571	268 (150, 543)	185 (100, 378)
HPV11L1	500	86 (334, 194)	37 (16, 83)
HPV16L1*	422	345 (58, 1715)	40 (18, 85)
HPV18L1	394	79 (41, 161)	49 (28, 97)
HPV31L1	712	100 (40, 293)	61 (29, 147)
HPV33L1	515	52 (27, 115)	40 (22, 76)
HPV35L1	552	95 (41, 285)	63 (34, 143)
HPV45L1	368	49 (24, 111)	33 (17, 64)
HPV52L1	547	67 (34, 183)	42 (22, 81)
HPV58L1	371	81 (42, 192)	58 (33, 107)
HPV6bE6	500	13 (5, 27)	12 (4, 24)
HPV11E6	260	39 (22, 64)	42 (23, 78)
HPV16E6*	484/1000	5204 (40, 9232)	14 (6, 28)
HPV18E6	243	10 (2, 25)	11 (1, 25)
HPV31E6	890	32 (15, 69.25)	25 (12, 60)
HPV33E6	253	34 (11, 118)	14 (3, 35)
HPV35E6	271	36 (19, 63)	26 (14, 45)
HPV45E6	249	10 (1, 24)	10 (3, 23)
HPV52E6	271	32 (11, 65)	25 (6, 52)
HPV58E6	250	33 (16, 74)	28 (14, 50)
HPV6bE7	364	6 (1, 16)	4 (1, 13)
HPV11E7	200	1 (1, 9)	1 (1, 6)
HPV16E7*	548	817 (26, 7608)	28 (10, 87)
HPV18E7	789	2 (1, 27.44)	1 (1, 20)
HPV31E7	200	31 (5, 1223)	7 (1, 21)
HPV33E7	500	110 (9, 2512)	10 (2, 29)
HPV35E7	384	69 (10, 1576)	14 (4, 29)
HPV45E7	200	2.3 (1, 15)	1 (1, 8)
HPV52E7	200	15 (2, 77)	6 (1, 20)
HPV58E7	200	29 (7, 260)	15 (6, 30)
HPV16E1*	200	322 (37, 2905)	21 (11, 36)
HPV16E2*	679	2885 (32, 8562)	24 (12, 52)
HPV16E4	876	240 (81, 1534)	98 (41, 277)
HPV18E1	200	51 (23, 239)	21 (11, 36)
HPV18E2	600	367 (75, 2919)	81 (49, 146)
HPV18E4	800	39 (21, 70)	36 (20, 64)

Calculations based on 1643 OPC cases and 1543 controls. Missing values: 3 for HPV16L1.

*HPV Markers involved in main analysis and/or calculation of HPV16 seropattern
MFI, Median Fluorescence Intensity

Supplementary Table 6: Suggested variants from HPV(+)OPC association results ($5x10^{-7} < P > 5x10^{-8}$).

Position	Locus	Variant	Chr:pos	Frequency (%)	Meta-analysis[a]	
				Controls	OR (95%CI)	P value
HPV16 positive						
3q26.1	609bp 5' of AC092965.1	rs9836282	chr3:166742591	48	0.76 (0.68-0.84)	2×10^{-7}
4q35.1	ODZ3 (intronic)	rs1516535	chr4:183255689	23	1.42 (1.25-1.63)	2×10^{-7}
HPV16 negative						
3p26.2	CNTN4 (intronic)	rs75622279	chr3:2905056	4.9	2.07 (1.59-2.71)	8×10^{-8}
1p36.13	7.9kb 5' of Y_RNA	rs12079362	chr1:17476763	2.6	3.94 (2.31-6.73)	5×10^{-7}
13q33.3	MYO16 (intronic)	rs55864736	chr13:109815112	27	1.44 (1.25-1.65)	4×10^{-7}
5q34	3.1kb 3' of MAT2B	rs729353	chr5:162949420	43	1.40 (1.24-1.59)	1×10^{-7}
6p21.32	3' UTR of HLA-DQA1	rs115210925	chr6:32614094	28	1.48 (1.27-1.71)	2×10^{-7}

OR, odds ratio; 95%CI, confidence interval

[a] Regional meta-analyses of European and North American GWAs association statistics

Supplementary Table 7: HPV(+) OPC associations results (<5x10^{-8}) and functional information based on public databases.

Significant_variants_OPC_HPVpositive	RegulomeBD score[a]	Haploreg	INFO annotation	UCSF Quimera	GTExv8	GWAs p-val [b]
rs4713462	1f	significant eQTL hit	MHC Intergenic	-	significant eQTL HLA-C	4.54E-11
rs4713460	5	significant eQTL hit	MHC Intergenic	-	significant eQTL HLA-C	6.08E-11
AA DRB1 71 E	4	missense_variant	HLA_DRB1 / rs9269942	protein-binding groove	-	2.79E-09
rs1049068	4	synonymous_variant	HLA_DQB1 coding region	-	significant eQTL HLA-DQB1	3.78E-09
rs12524487	6	significant eQTL hit	MHC Intergenic	-	significant eQTL HLA-C /MICA	5.26E-09
HLA DRB1 13	-	-	pool of HLA-DRB1*13 proteins	-	-	7.36E-09
rs41547417	4	transcript variant/missense variant	HLA DQA1 coding region	-	-	8.67E-09
HLA DQA1 0103	-	-	protein		-	8.69E-09
rs9266144	4	transcript variant/missense variant	HLA B coding region	-	-	1.04E-08
AA B 156 W	4	missense variant	rs697742	protein-binding groove	-	1.05E-08
HLA B 1501	-	-	protein		-	1.13E-08
HLA DRB1 1301	-	-	-		-	1.44E-08
AA DRB1 74 A	4/4	frameshift variant/missense	rs67476479 / rs17886882		-	2.35E-08
HLA DQB1 0603	-	-	protein		-	3.08E-08
rs12528645	4	HLA-B intronic	HLA B		-	3.11E-08
rs12526858	4	HLA-B intronic	HLA B		significant eQTL HLA-C /MICA	3.14E-08
rs4999717	4	HLA-B intronic	HLA B		significant eQTL HLA-C /MICA	3.24E-08
rs1131161	4	synonymous variant	HLA B coding region		-	3.29E-08
AA DRB1 37 F	4	missense variant	HLA DRB1 / rs17883134	other position	-	3.69E-08
rs17193012		HLA-B intronic	-		-	3.75E-08
rs3819294	4	HLA-B intronic	-	-	-	3.90E-08
rs9266688	no data	some regulatory evidence	MHC Intergenic		significant eQTL HLA-C /MICA	3.97E-08
HLA B 15	-	-	pool of HLA-DRB1*15 proteins	-	-	3.99E-08

rs9380254	no data	missense variant	MICA	-	significant eQTL HLA-C /MICA	4.26E-08
rs4293988	6	some regulatory evidence	MHC Intergenic	-	significant eQTL HLA-C /MICA	4.55E-08
rs9266722	5	some regulatory evidence	MHC Intergenic	-	significant eQTL HLA-C /MICA	4.74E-08

[a]The Regulome BD scoring refers to the DNA features and regulatory regions evidence being 1a the highest level of evidence and 6 the lowest.

[b] Regional meta-analyses of European and North American GWAs association statistics

Supplementary table 8: HPV(+)OPC association results for the other *HLA-DRB1* alleles containing amino acid 71-Glu as part of their sequence.

HLA-DRB1 variant	Info	Frequency (%) Controls / Cases	Meta-analysis[b] OR (95%CI)	P value	Q_p
Top associated in HLA-DRB1					
**13:01*	0.99	6.7 / 2.8	0.43 (0.32-0.58)	1.44×10^{-8}	0.95
71-Glu[a]	0.94	14.5 / 7.9	0.56 (0.47-0.68)	2.80×10^{-9}	0.30
Other HLA-DRB1 containing amino acid 71-Glu					
**04:02*	0.91	1.5 / 0.4	0.37 (0.17-0.84)	0.02	0.84
**13:02*	0.99	4.2 / 2.9	0.72 (0.54-0.96)	0.03	0.89
**11:02*	0.81	0.3 / 0.1	0.45 (0.09-2.26)	0.34	0.49

OR, odds ratio; 95%CI, confidence interval

[a] amino acid change rs9269942 C/A [Ala (GCG) --> Glu (GAG)]

[b] Regional meta-analyses of European and North American GWAs association statistics

Supplementary table 9: HPV16 seropattern in OPC cases and controls stratified by other less frequent HPV(+)OPC definitions and by HPV16 E6 status.

OPC HPV-driven definitions	OPC		Controls	
	HPV16 seropattern+	HPV16seropattern -	HPV16 seropattern+	HPV16seropattern -
High risk HPV E6 & E7, not HPV16 [a,b]				
positives	183	30	0	2
negative	894	532	16	1525
HPV16 E6				
positives	1059	565	15	0
negative	19	0	1	1527
N total	1078	565	16	1527

a Additional HPV(+)OPC definition based on E6 & E7 positivity of other high risk HPV types (18, 31, 33, 35, 45, 52 and 58)

b Missing value: 4 subjects

Supplementary table 10: Association results of HPV(+)OPC top hits using other OPC HPV-driven definitions.

OPC HPV-driven definitions	Variant	Frequency (%) Controls / Cases	OR (95%CI)[g]	P value[g]
High risk HPV E6 & E7, not HPV16 [a,b]				
Positive cases vs controls[c]				
	rs4713462	32.6 / 21.2	0.64 (0.36-1.14)	0.13
	HLA-DRB1 (Glu-71)	14.5 / 15.2	1.16 (0.58-2.33)	0.66
Negative cases vs controls[d]				
	rs4713462	32.6 / 30.7	0.99 (0.58-2.33)	0.9
	HLA-DRB1 (Glu-71)	14.5 / 10.1	0.71 (0.58-0.88)	0.002
HPV16 E6				
Positive cases vs controls[e]				
	rs4713462	32.6 / 20.8	0.62 (0.55-0.70)	5.3×10^{-13}
	HLA-DRB1 (Glu-71)	14.5 / 7.7	0.52 (0.44-0.63)	1.5×10^{-11}
Negative cases vs controls[f]				
	rs4713462	32.6 / 30.3	0.98 (0.85-1.11)	0.73
	HLA-DRB1 (Glu-71)	14.5 / 10.4	0.74 (0.60-0.90)	0.003

OR, odds ratio; 95%CI, confidence interval

a HPV types 18, 31, 33, 35, 45, 52 and 58; excluding those HPV16 seropattern positive

b Missing value: 4 subjects

c OPC Other High risk types Positive (N=33) vs controls (N=5262)

d OPC Other High risk typesNegative (N=574) vs controls (N=5262)

e OPC HPV16 E6 Positive (N=1059) vs controls (N=5262)

f OPC HPV16 E6 Positive (N=565) vs controls (N=5262)

g [a] Obtained from multivariate logistic regression assuming an additive genetic model with sex and principal components as covariates

Supplementary Table 11: HPV(+)OPC top genome-wide significant regions and their most likely functional variants from regional meta-analyses of oropharyngeal cancers by HPV16 seropattern status as judged by the BIC criterion.

HPV(+)OPC Independent regions	Locus	Variant	P value[a]	BIC[a]	BIC dif[a,b]
HLA class I					
	Intergenic	rs4713462	4.5×10^{-11}	4486	0
	HLA-B	*1501*	1.13×10^{-8}	4516	+30
		156 -Trp	1.05×10^{-8}	4516	+30
HLA class II					
	Class II haplotype	DRB1*13:01-DQA1*01:03-DQB1*06:03	7.4×10^{-9}	4500	+13
	HLA-DRB1	*1301*	1.44×10^{-8}	4501	+14
		71 -Glu[c]	2.8×10^{-9}	4487	+1
		74 -Ala	2.6×10^{-8}	4494	+7
	HLA-DQA1	*0103*	8.7×10^{-9}	4495	+8
	HLA-DQB1	*0603*	3.1×10^{-8}	4504	+17

BIC, Bayesian information criterion; OR, odds ratio; 95%CI, confidence interval

[a] Obtained from multivariate logistic regression assuming an additive genetic model with sex and principal components as covariates

[b] Models having their BIC difference within: +1–2 of the minimum have substantial support; + 4–7 of the minimum have considerably less support; BIC >10 above the minimum fail to explain some substantial structural variation in the data.

[c] Alleles where these amino acids are part of the sequence: 71(Glu) in *DRB1**13:01 and also in *04:02, *13:02 and *11:02.

Supplementary Table 12: Extended results on model selection from HLA alleles, SNP and amino acids from HPV(+)OPC analyses as judged by the BIC criterion.

	Model	Locus	OR (95%CI)[a]	P value	BIC	BIC dif[b]
A	rs4713462 + DRB1 (71-glu)				4454	0
		rs4713462	0.67 (0.59-0.76)	1.5×10^{-10}		
		DRB1 (71-glu)[c]	0.55 (0.46-0.67)	1.0×10^{-9}		
B	HLA-B*1501 + DRB1 (71-glu)				4475	+21
		HLA-B*15:01	0.52 (0.39-0.69)	8.7×10^{-6}		
		DRB1 (71-glu)[c]	0.53 (0.43-0.63)	1.0×10^{-11}		
	B (156) + DRB1 (71-glu)				4474	+21
		B (156)	0.52 (0.40-0.70)	7.5×10^{-6}		
		DRB1 (71-glu)[c]	0.53 (0.43-0.63)	1.5×10^{-11}		
C	rs4713462 + DRB1 (74-ala)				4455	+1
		rs4713462	0.65 (0.58-0.74)	1.3×10^{-10}		
		DRB1 (74-ala)	0.67 (0.60-0.76)	2.5×10^{-9}		
	HLA-B*1501 + DRB1 (74-ala)				4483	+29
		HLA-B*15:01	0.53 (0.40-0.71)	2.3×10^{-05}		
		DRB1 (74-ala)	0.67 (0.59-0.75)	7.9×10^{-10}		
	B (156) + DRB1 (74-ala)				4483	+29
		B (156)	0.54 (0.41-0.72)	2.2×10^{-05}		
		DRB1 (74-ala)	0.68 (0.59-0.75)	8.3×10^{-10}		
D	rs4713462 + Class II Haplotype				4465	+11
		rs4713462	0.66 (0.58-0.75)	5.5×10^{-11}		
		DRB1*13:01-DQA1*01:03-DQB1*06:03	0.45 (0.34-0.61)	1.1×10^{-7}		
	HLA-B*1501 + Class II Haplotype				4489	+35
		HLA-B*15:01	0.53 (0.40-0.71)	1.8×10^{-05}		
		DRB1*13:01-DQA1*01:03-DQB1*06:03	0.43 (0.32-0.58)	3.2×10^{-8}		
	B (156) + Class II Haplotype				4489	+35
		B (156)	0.54 (0.41-0.71)	1.5×10^{-05}		
		DRB1*13:01-DQA1*01:03-DQB1*06:03	0.43 (0.32-0.58)	3.1×10^{-8}		
E	rs4713462 + DRB1 (71-glu)+ DRB1 (74-ala)				4447	-7
		rs4713462	0.67 (0.59-0.76)	4.8×10^{-10}		
		DRB1 (71-glu)[c]	0.61 (0.50-0.74)	6.7×10^{-6}		
		DRB1 (74-ala)	0.72 (0.64-0.82)	4.2×10^{-6}		

BIC, Bayesian information criterion; OR, odds ratio; 95%CI, confidence interval

[a] Obtained from multivariate logistic regression assuming an additive genetic model with sex and principal components as covariates

[b] Models having their BIC difference within: +1–2 of the minimum have substantial support; + 4–7 of the minimum have considerably less support; BIC >10 above the minimum fail to explain some substantial structural variation in the data.

[c] Alleles where these amino acids are part of the sequence: 71(Glu) in *DRB1**13:01 and also in *04:02, *13:02 and *11:02.

Supplementary table 13: Linear models on log-transformed HPV16 E6 (A) and L1 MFI levels (B) exploring HLA genetic variants association by adjusting for the alternative serology marker in overall OPC (N=1643). In addition, all models were adjusted by age, sex and eigenvectors. MFI, Median Fluorescence Intensity

A

Regression Model on E6 MFI levels			
Model	Variants	B	P value
A	DRB1 71-Glu	-0.12	0.11
B	rs4713462	-0.31	1.3×10^{-8}
C	L1 MFI levels	0.81	$<10^{-16}$
D	rs4713462 + L1 MFI levels		
	rs4713462	-0.27	8.1×10^{-8}
	L1 MFI levels	0.8	<10-16

B

Regression Model on L1 MFI levels			
Model	Variants	B	P value
A	rs4713462	-0.07	0.05
B	DRB1 71-Glu	-0.30	7.0×10^{-8}
C	E6 MFI levels	0.37	$<10^{-16}$
D	DRB1 71-Glu + E6 MFI levels		
	DRB1 71-Glu	-0.31	3.1×10^{-11}
	E6 MFI levels	0.36	$<10^{-16}$

Supplementary Figure 1: Quantile-Quantile Plots for regional meta-analysis of GWAs, (a) OPC HPV16 positive, (b) OPC HPV16 negative, (c) OC excluding South America from Lesseur et al NatGen 2016 and (d) pooled OPC HPV16 negative and OC. All analyses were obtained from multivariate unconditional logistic regression assuming an additive genetic model with sex, age and eigenvectors as covariates.

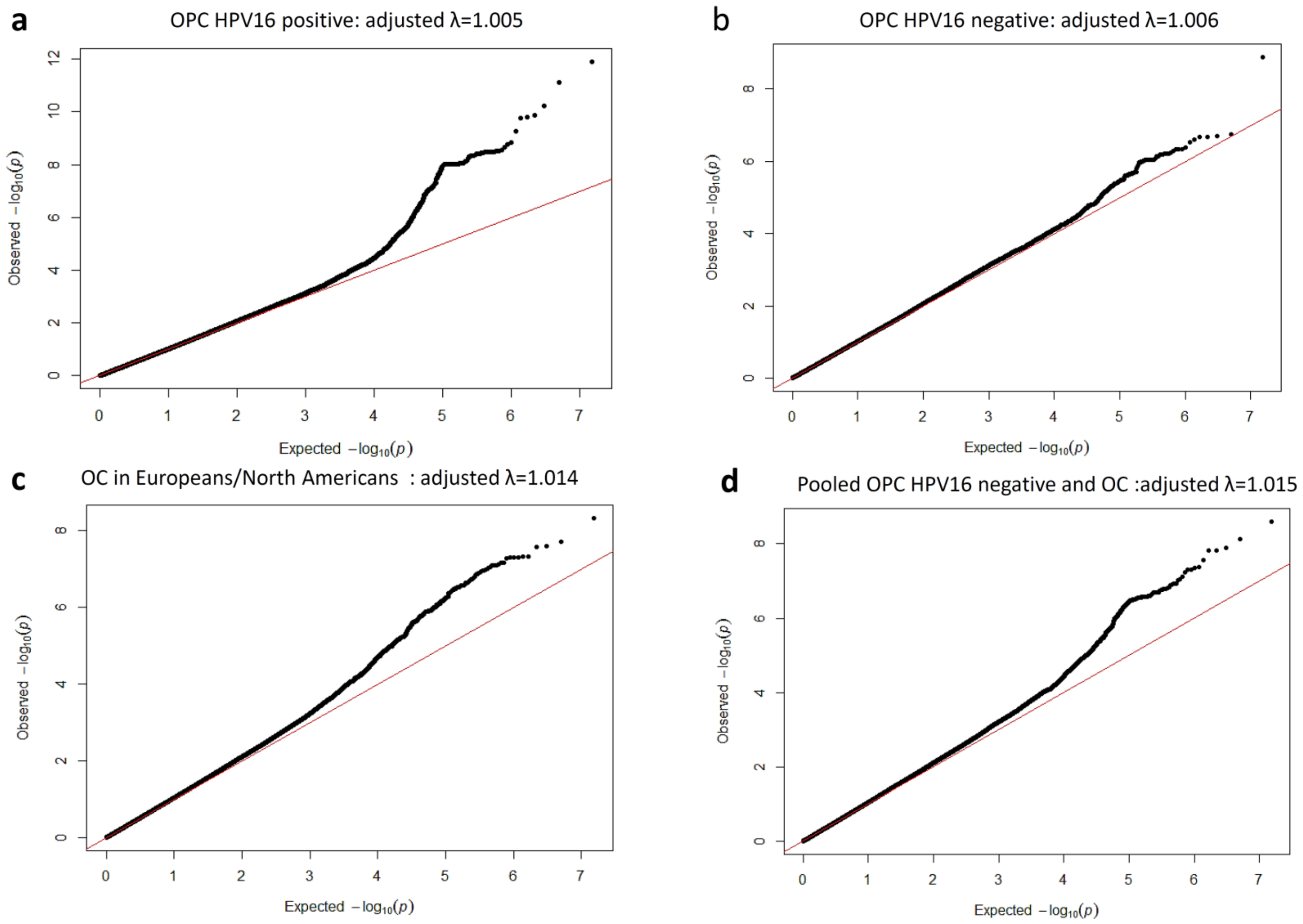

Supplementary Figure 2: Stratified analyses of top-ranking variants for OPC HPV16 positive (a-c) and OPC HPV16 negative (d). Results are shown for (a) the HLA Class II haplotype (b) *HLA-DRB1 71-Glu*, (c) *rs4713462*, (d) *rs35189640*. Stratified analyses by histology and smoking status were obtained from multivariate unconditional logistic regression assuming an additive genetic model with sex, age and eigenvectors as covariates; stratified analyses by sex included just age and eigenvectors as covariates. Discovery samples correspond to those with HPV information in Lesseur at al.2016.

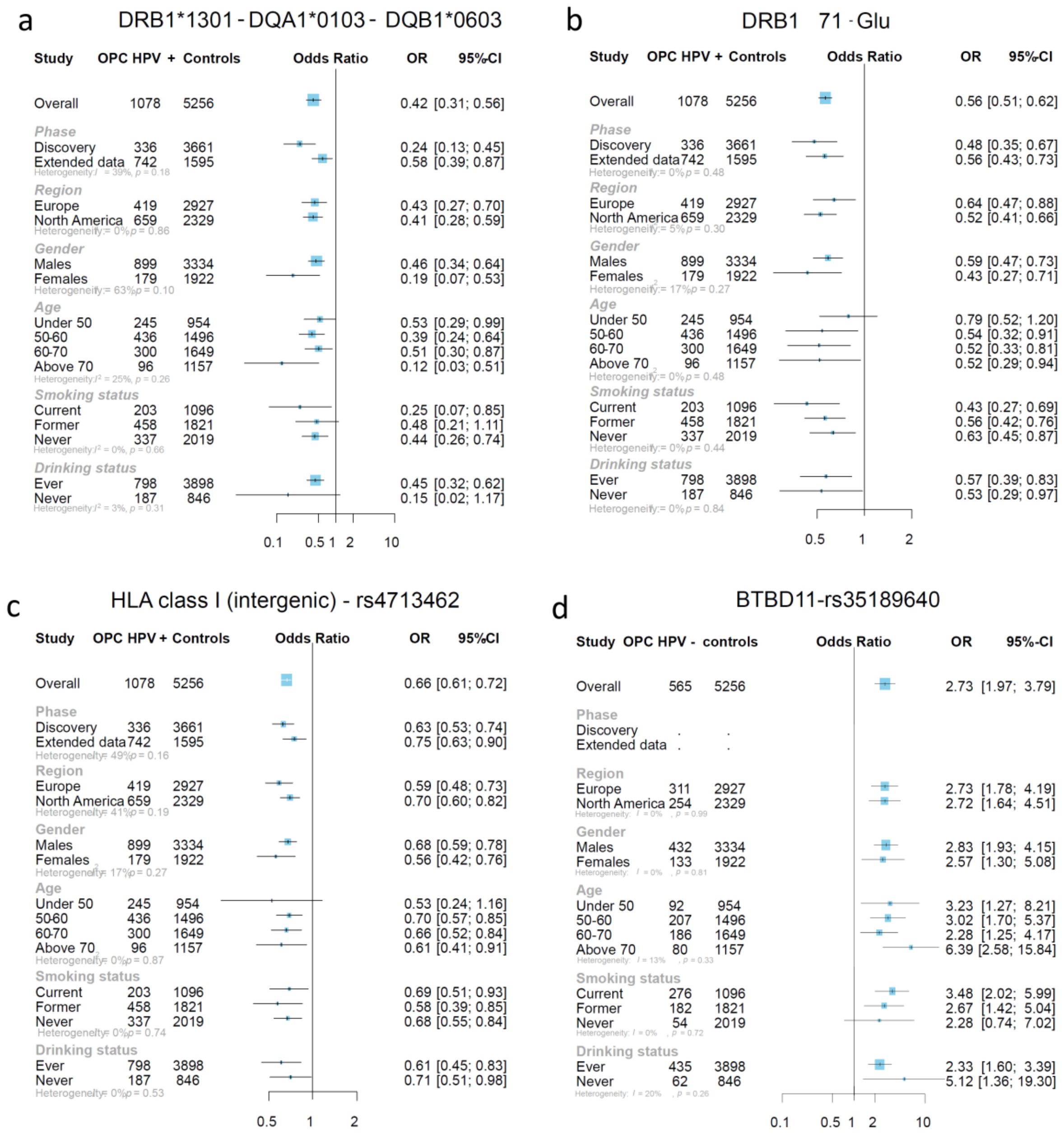

Supplementary Figure 3: Scatter plots comparing for variants across the MHC region, their OPCHPV(+) association results (z-score) (Y-axis) with their skin-related eQTL association results (z-score) (GTEx v8) (X-axis). Each variant (dot) is colored relative the degree of linkage disequilibrium (r2) with sentinel OPC HPV(+) variant (rs4713462).

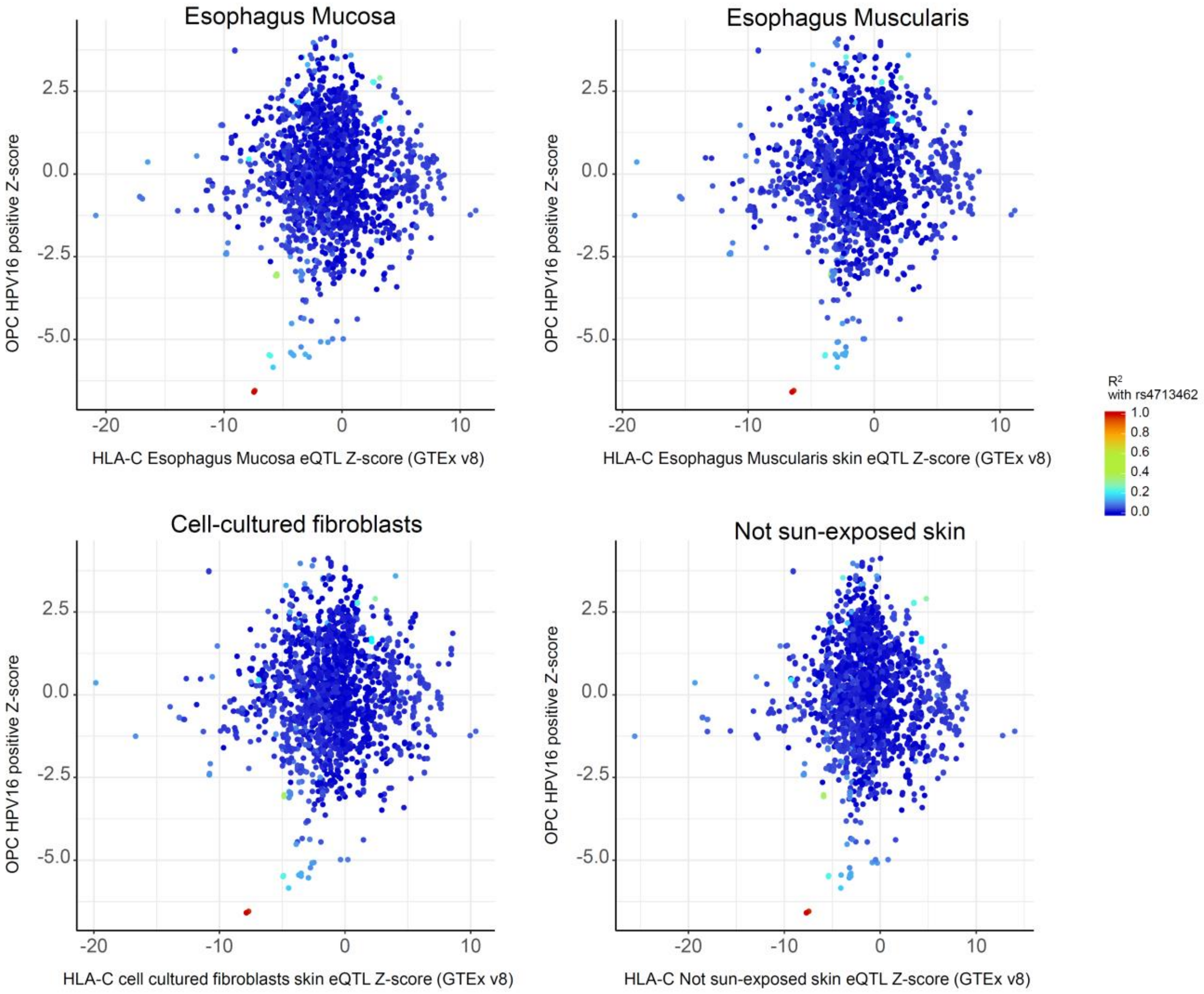

Supplementary Figure 4: Three-dimensional ribbon models for the HLA-B (a) and HLA-DR (b) proteins. These structures are based on Protein Data Bank entries 2bvp and 3pdo, respectively, with a direst view of the peptide-binding groove. Key amino acid positions identified in OPV HPV16 positive association analyses are highlighted. This figure was prepared using UCSF Chimera. Association analyses were obtained from multivariate unconditional logistic regression assuming an additive genetic model with sex, age and eigenvectors as covariates.

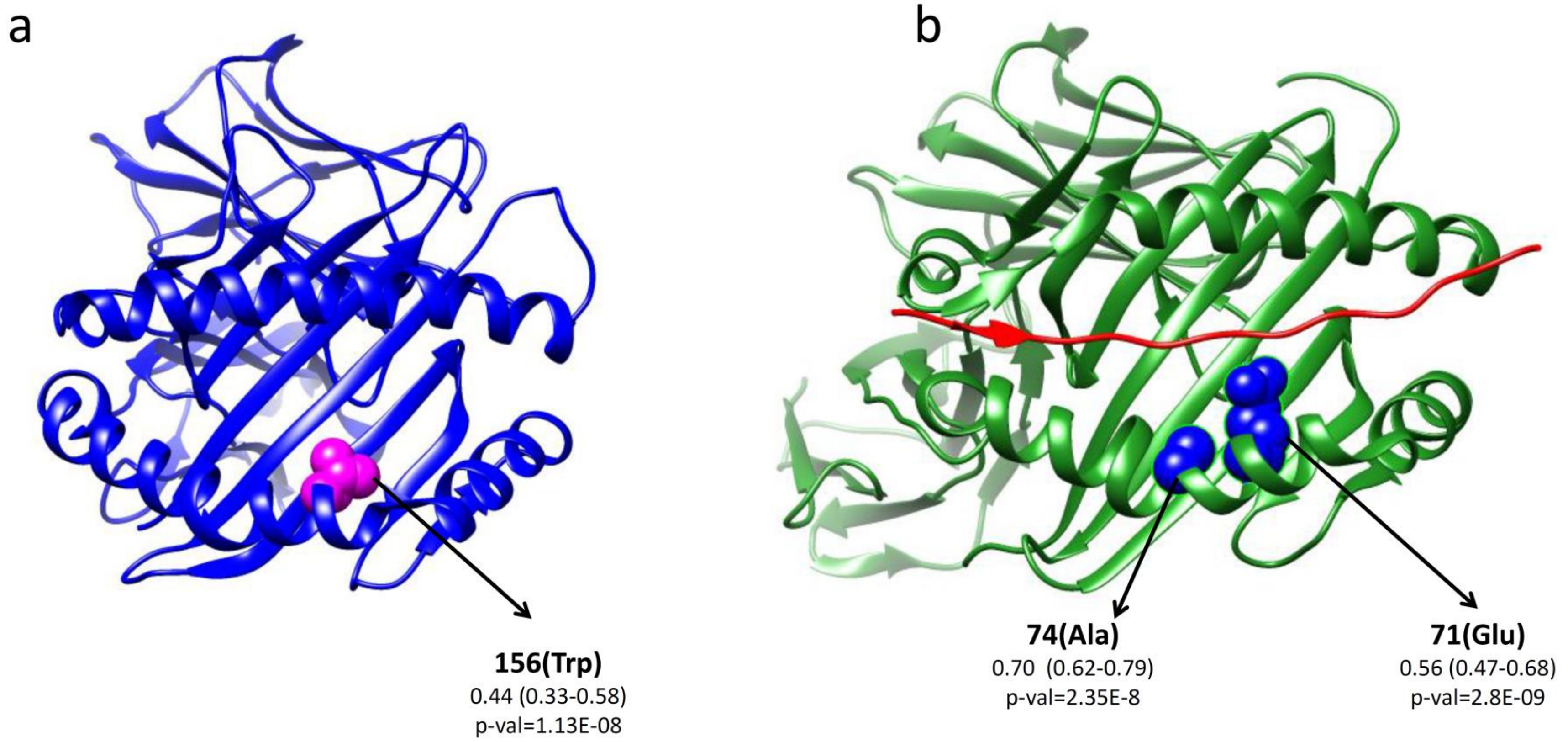

Supplementary Figure 5: Regional plot of OPC HPV negative GWAs top hit in chromosome 12 (BTBD11 gene) with functional annotation panels.(a) GWAS association statistics obtained from multivariate unconditional logistic regression assuming an additive genetic model with sex, age and eigenvectors as covariates, (b)CADD score and RegulomeDB score (c) the annotations of core 15 chromatin states profiled by ChromHMM; Chromatin states were evaluated in more than 30 tissues and the plot is displaying those with meaningful result. Each row corresponds to one of the cell or tissue types from different anatomy regions (ESC, Embryonic stem cell). Chromatine states: [active TSS (TssA), flanking active TSS (TssAFlnk), transcription at gene 5′ and 3′ (TxFlnk), strong transcription (Tx), weak transcription (TxWk), genic enhancers (EnhG), enhancers (Enh), ZNF genes & repeats (ZNF/Rpts), heterochromatin (Het), bivalent/poised TSS (TssBiv), flanking bivalent TSS/Enh (BivFlnk), bivalent enhancer (EnhBiv), repressed PolyComb (ReprPC), weak repressed PolyComb (ReprPCWk), and quiescent/low (Quies)]. No eQTL of the studied tissues was found in this region.

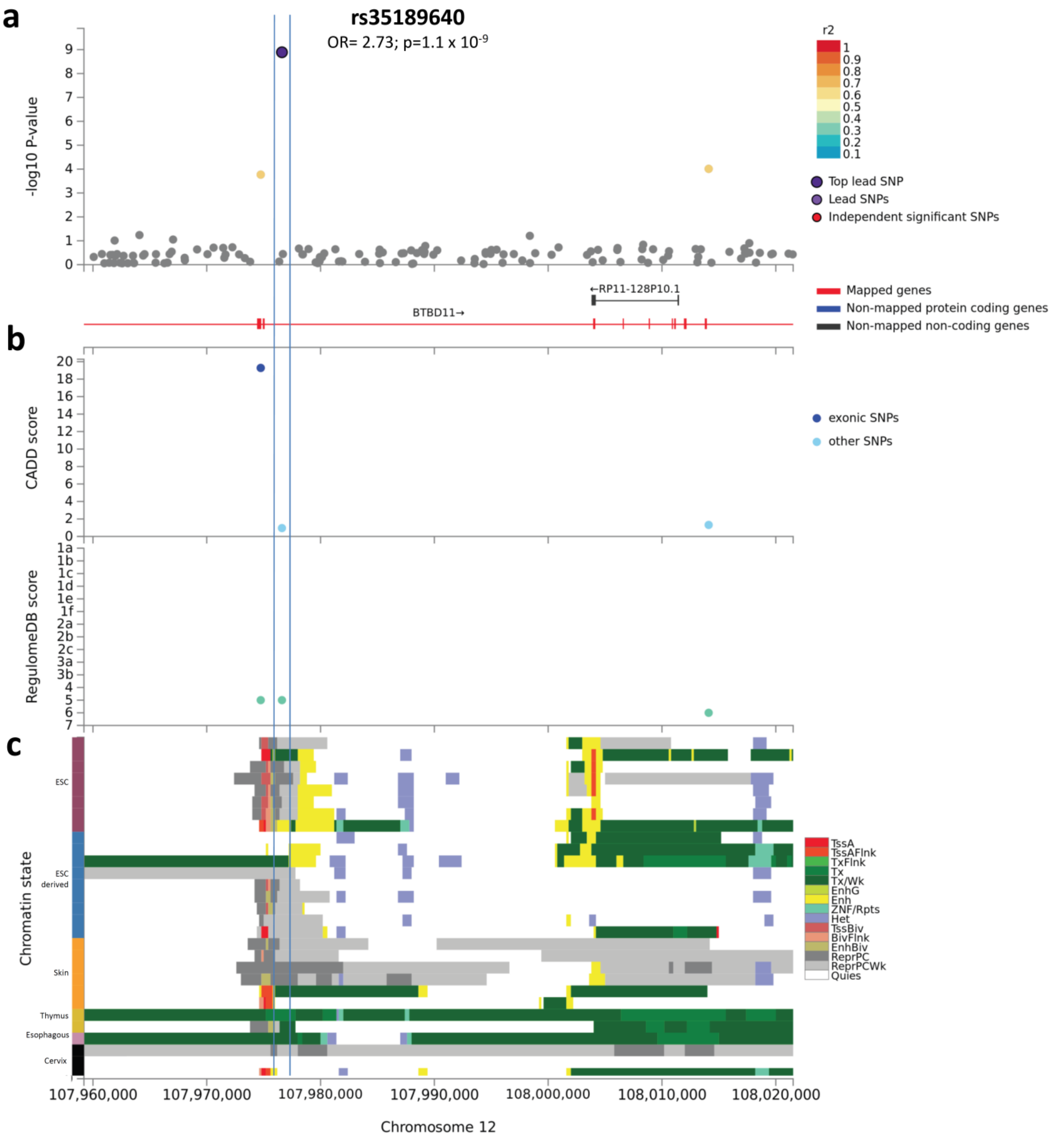

Supplementary figure 6: pQTL analysis of plasma antibody levels against HPV16 E2 and E7 proteins of the top two associated OPC HPV(+) HLA variants. Box plots showing HPV16 E2 MFI levels and rs4713462 or HLA-DRB1 (71-Glu) genotypes (top panel); and HPV16 E7 MFI levels and rs4713462 or HLA-DRB1 (71-Glu) genotypes (lower panel) among OPC cases. P-values were obtained from linear multivariate models implemented to test associations of genetic variants with each HPV type log-transformed MFI levels adjusted for age, sex and eigenvectors. For HLA-DRB1 71-Glu, A stands for absence and P stands for presence. Box plots show the medians (centre lines) and the 25th and 75th percentiles (box edges), with whiskers extending to 1.5 times the interquartile range. Sample size by genotype group was as follows for DRB1 71-Glu (AA=1369; AP=265; PP=6) and for rs4713462 (GG=951; GA=592; AA=100). MFI, Median Fluorescence Intensity

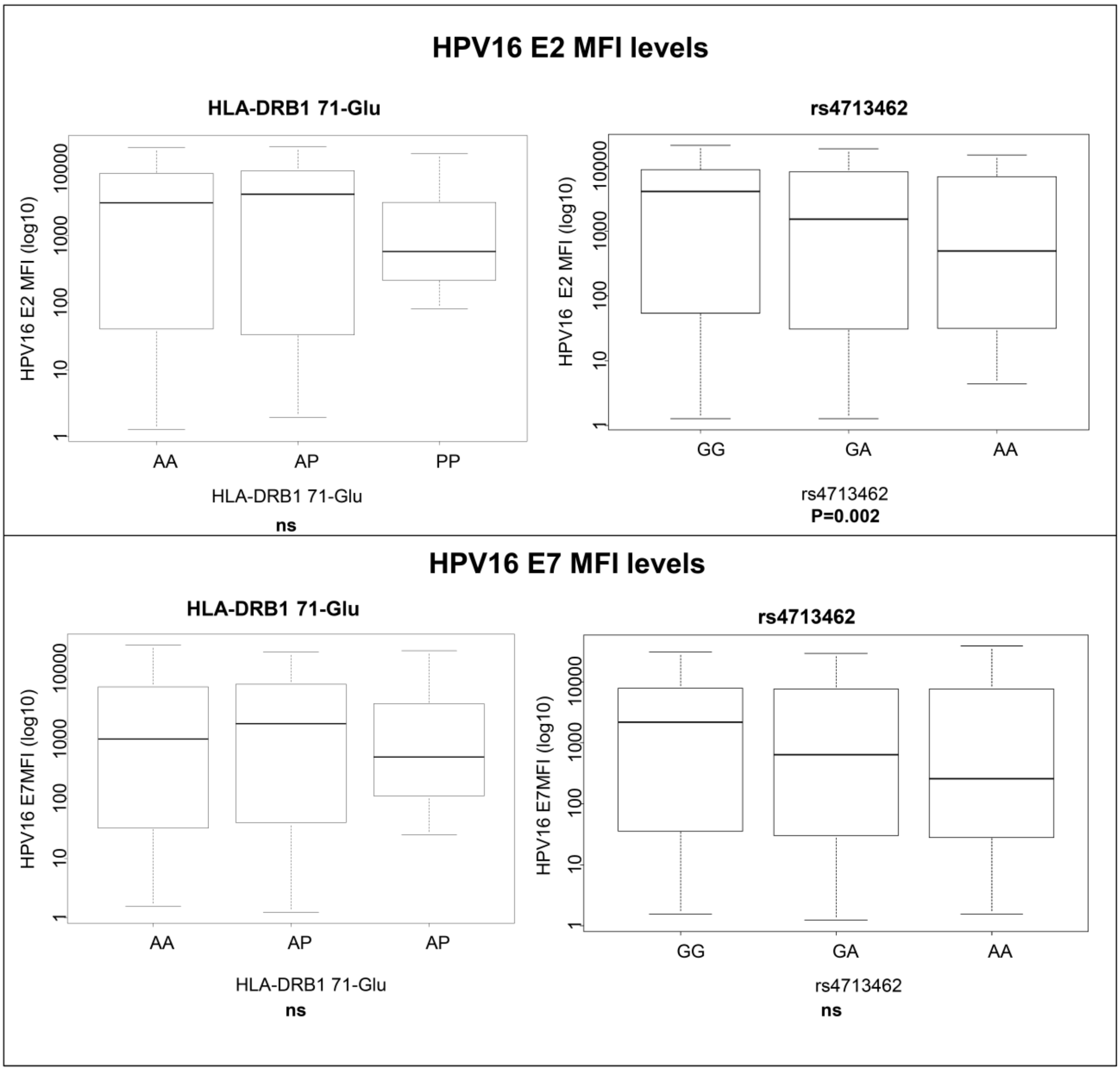

Supplementary Figure 7: Pooled HPV(-)OPC and OC genome-wide association results. Red and blue lines correspond to P = 5 × 10−8 and P=0.05, respectively. The y axes show −log10 P values. (a) Miami plot comparing genetic associations of (a) OC cancer cases analysis with 2,359 cases and 5,256 controls and (b) OPC HPV(-) cases analysis with 565 cases and 5,256 controls. (c) Pooled OPC HPV(-) and OC cancer cases analysis with a total of 2,924 cases and 5,256 controls. GWAs for each world region were performed using multivariable unconditional logistic regression assuming a log-additive genetic or dosage model with age, sex and eigenvectors as covariates. P-values are shown from fixed-effect meta-analysis of regional association statistics.

a

b

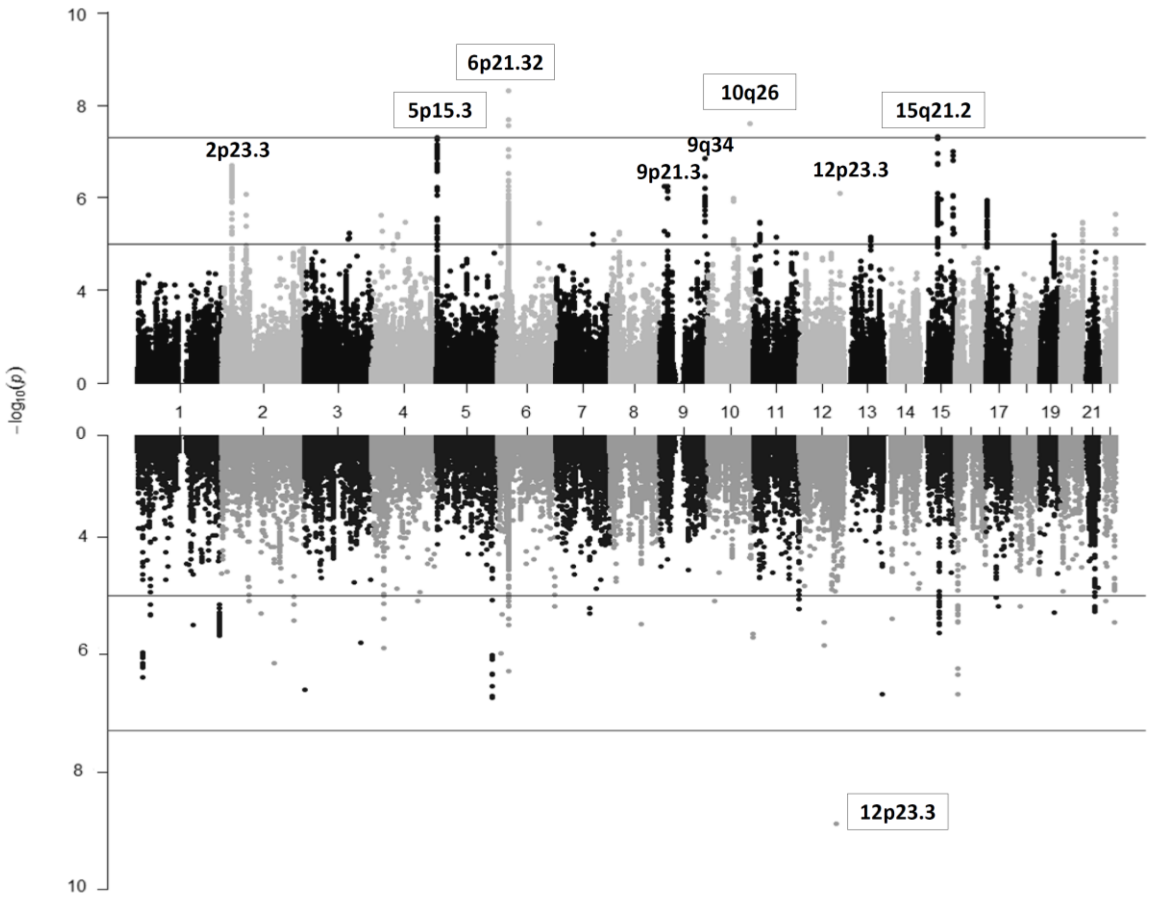

c

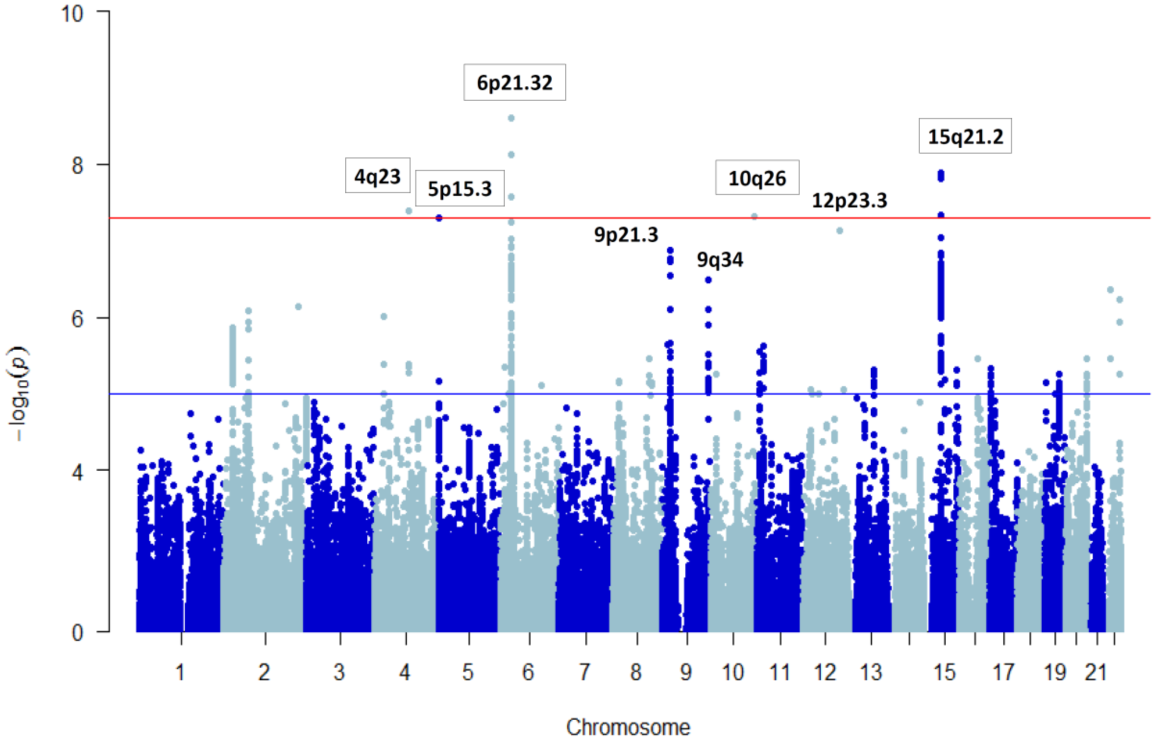

Supplementary Figure 8: Scatter plots comparing for all variants across the 15q21.2 locus, their OPCHPV(-)&OC association results (z-score) (Y-axis) with their epithelial-related cis eQTL association results (z-score) (GTEx v8) (X-axis). Effect sizes obtain either from logistic (Y-axis) or linear(X-axis) regression were adjusted for age, sex and eigenvectors. Each variant (dot) is colored relative the degree of linkage disequilibrium (r2) with sentinel OPCHPV(-)&OC variant (rs12910284). The rs12910284 tend to be cis-eQTL for FGF7 in fibroblasts but not in esophagus mucosa, esophagus muscularis and not sun-exposed skin.

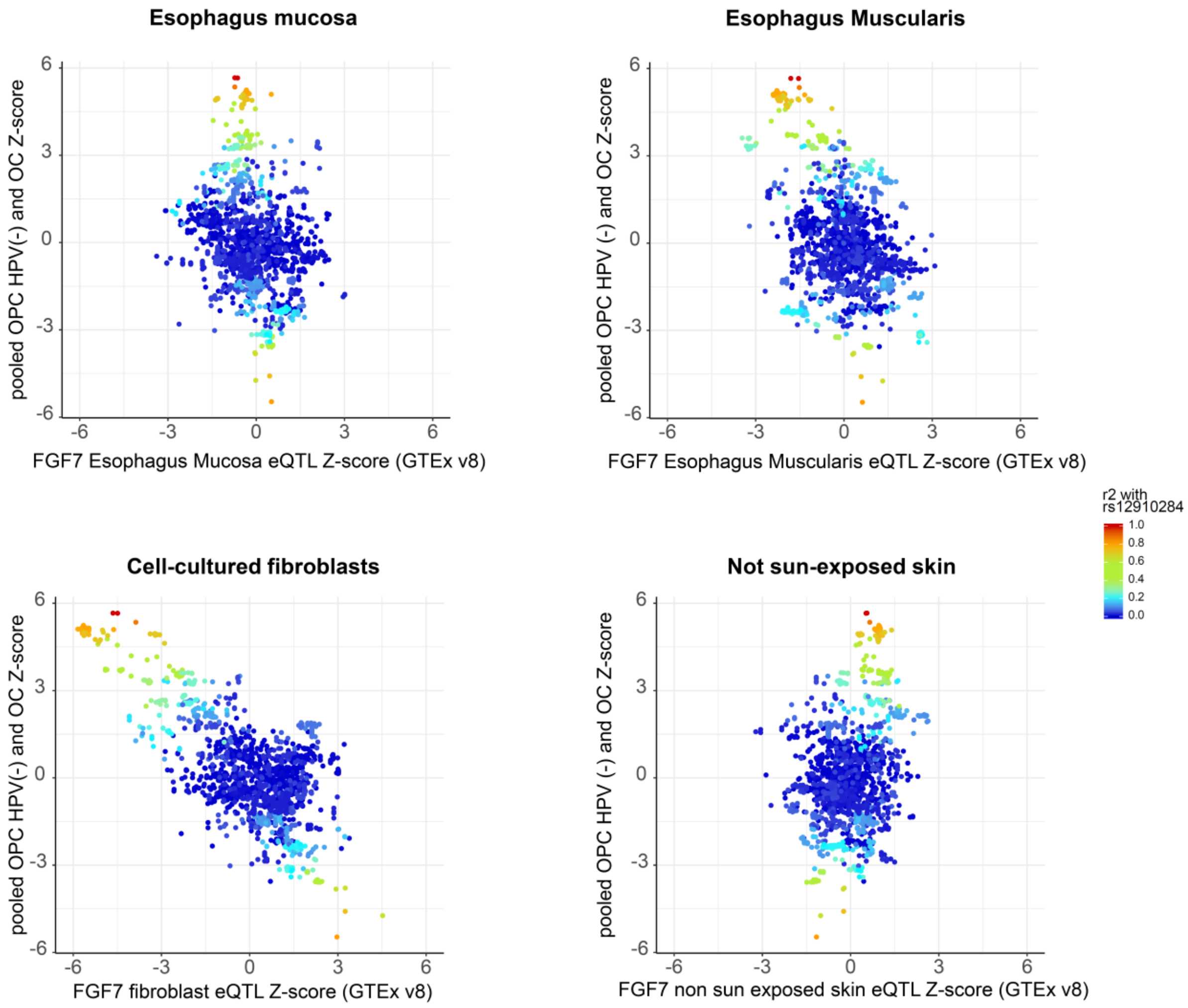

Supplementary Figure 9: OPC pQTL analysis of plasma antibodies levels against HPV proteins across MHC region (6p21.3 OPC). Scatter plots comparing, for all variants across the 6p21.3 locus , their HPV(+)OPC GWA association results (Z-score) (Y-axis), with their HPV16 E6 MFI (a) or L1 MFI (b) pQTL z-scores (X-axis) in OPC cases. Effect sizes obtain either from logistic (Y-axis) or linear(X-axis) regression were adjusted for age, sex and eigenvectors. Each variant (dot) is colored relative the degree of linkage disequilibrium (r2) with HPV(+)OPC top associated variants at 6p21.3. The top variants associated with HPV(+)OPC, rs4713462 and DRB1 Glu-71, also tend to be those that are the top OPC pQTLs for HPV16 E6 and L1 plasma antibody levels, respectively.

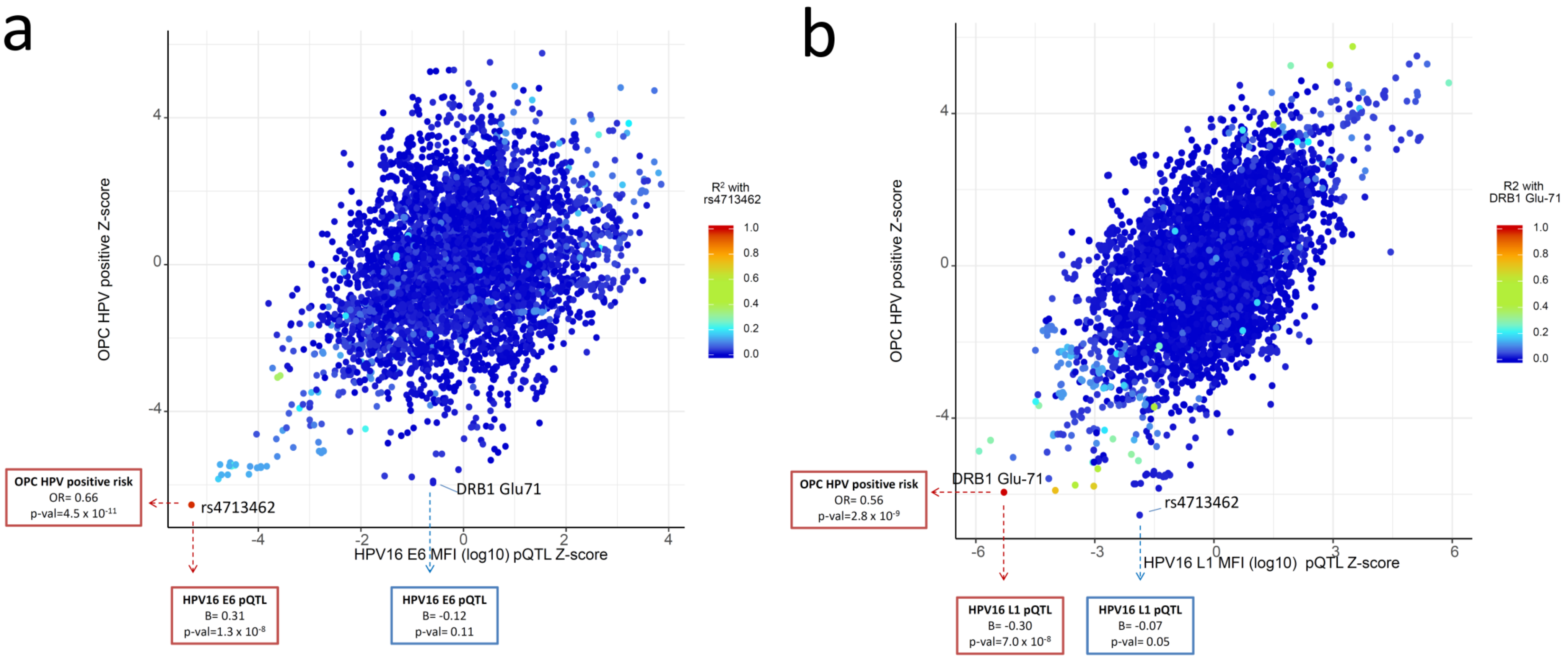

Supplementary Figure 10: Evaluation of the consistency of the top hits effects. Forest plots showing top hits across GWAs from a meta-analysis of (a) [HPV(+) and HPV(-)OPC] results or (b) [HPV(-)OPC and OC] after dividing the control group into two equivalent random series.

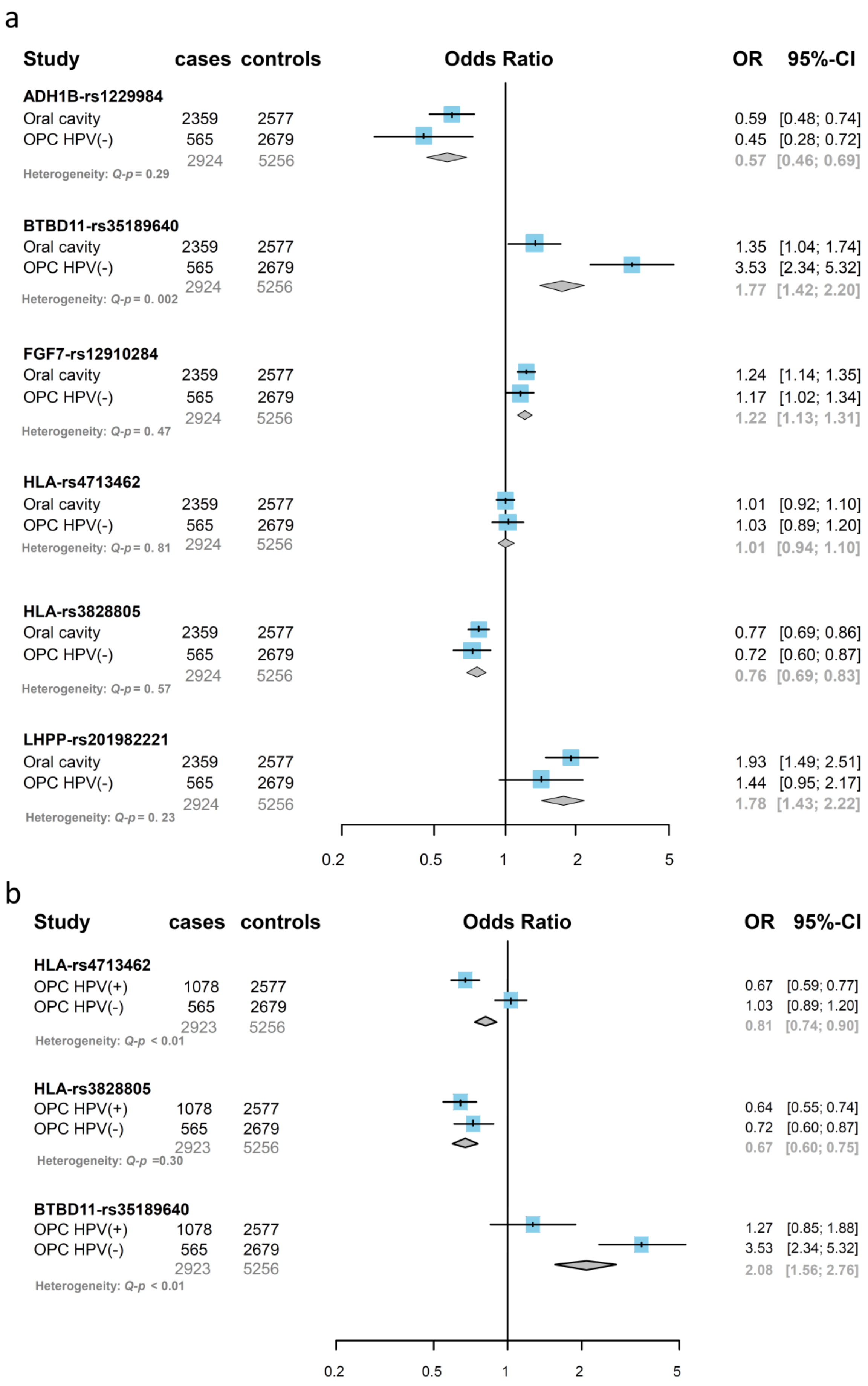

Supplementary Figure 11: pQTL analysis of plasma antibody levels against HPV proteins of the top two associated OPC HPV(+) HLA variants. Box plots showing (a) HPV16 E6 MFI levels (y axis) and rs4713462 genotypes (x axis), and (b) HPV16 L1 MFI levels(y axis) and DRB1 (71-Glu) genotypes (x axis) in OPC positive cases for each serology marker. Effect sizes (B, regression coefficient) obtain from linear regression were adjusted for age, sex and eigenvectors were adjusted for age, sex and eigenvectors. For HLA-DRB1 71-Glu, A stands for absence and P stands for presence. Box plots show the medians (centre lines) and the 25th and 75th percentiles (box edges), with whiskers extending to 1.5 times the interquartile range. MFI, Median Fluorescence Intensity

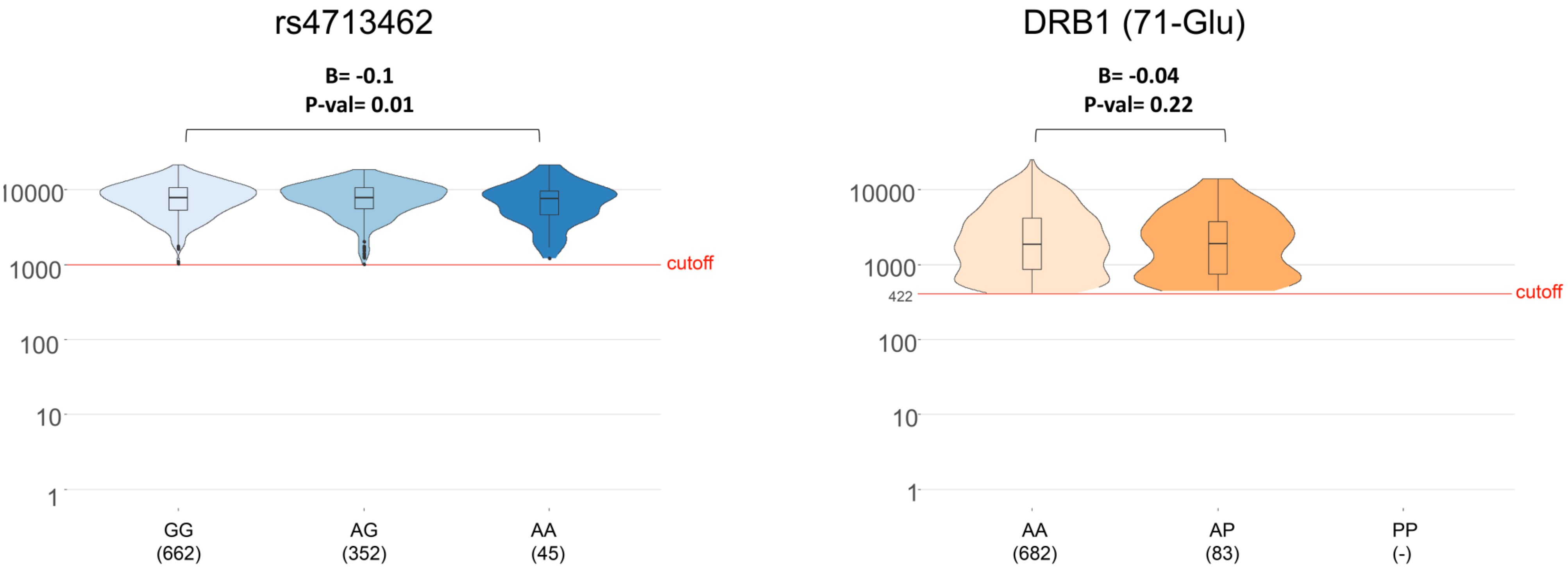

Supplementary Figure 12: Pairwise D′ and R^2 values plotted against genomic distance in HLA B and C region with rs4713462 position highlighted in the reference European population from Phase 3 of the 1000 Genomes Project (1000G).

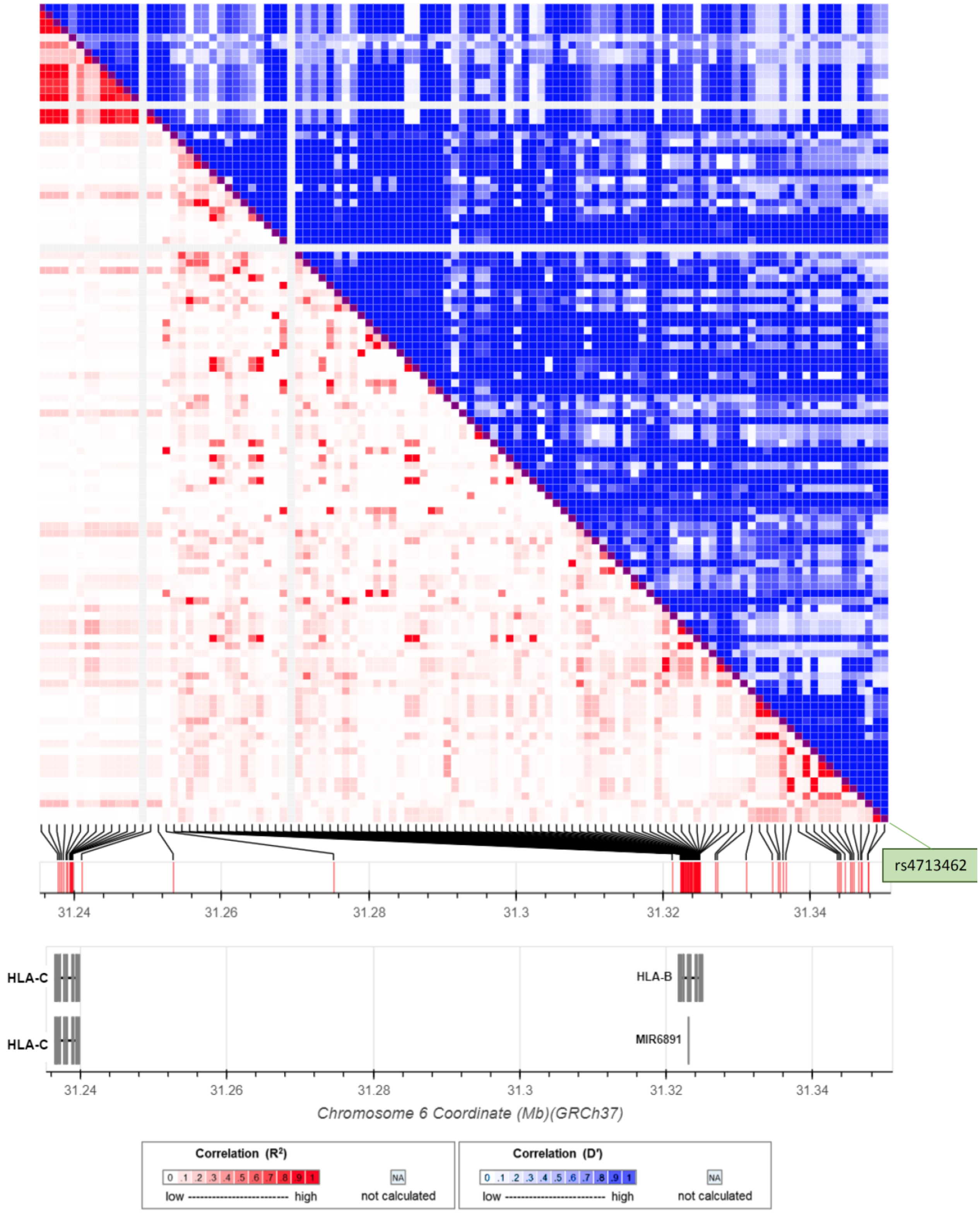

Supplementary Figure 13: Regional meta-analyses association plot of HLA-C and B including the rs4713462 that passed the genome-wide significance threshold, showing genes located at or near the significant loci, the extent of LD within the selected region and the functional annotation by Regulome BD. Regional meta-analyses GWAs association statistics obtained from multivariate logistic regression assuming an additive genetic model with sex and principal components as covariates

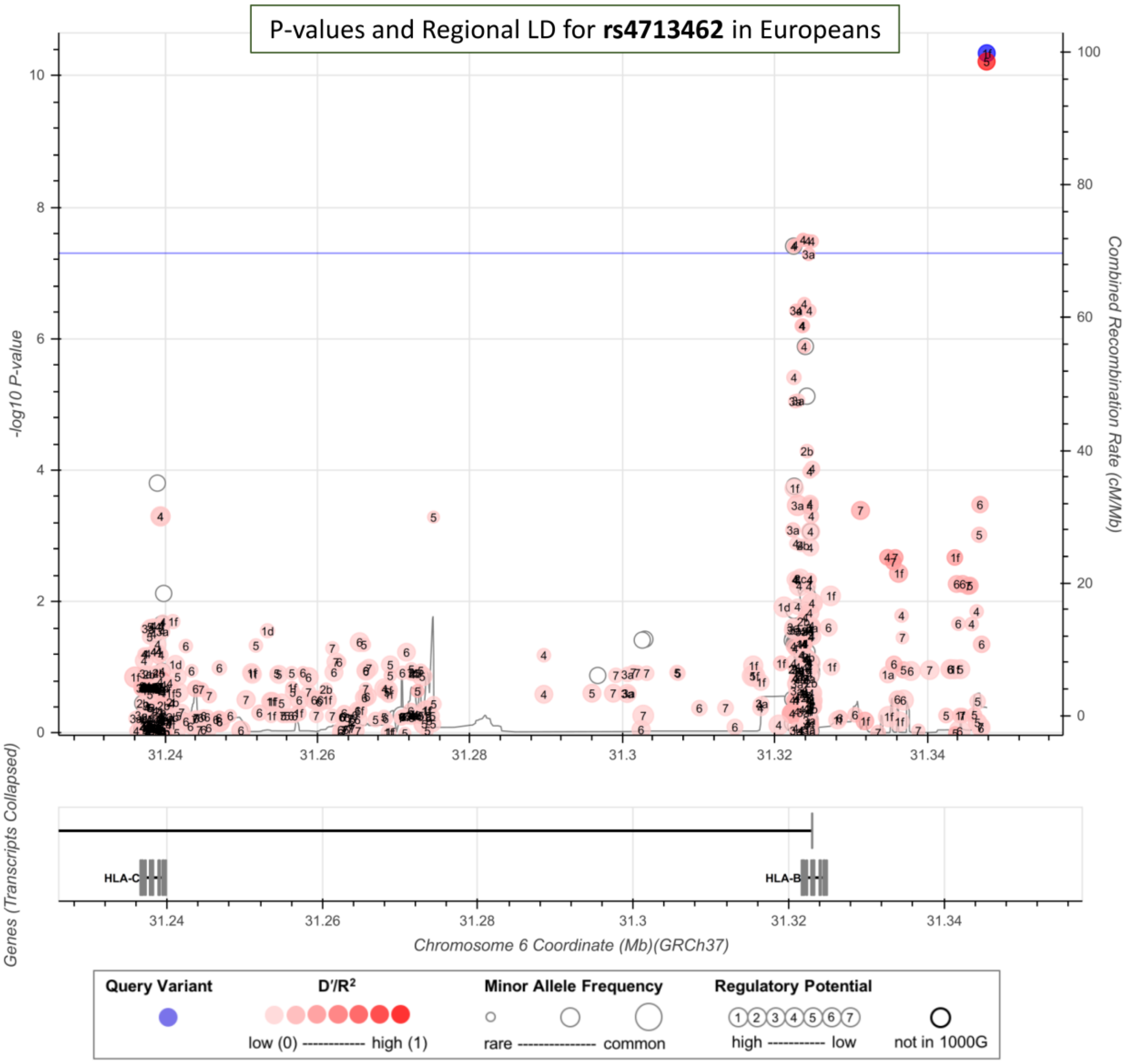

Supplement: Supplementary file 1 — Supplementary Information [file 41467_2021_26151_MOESM1_ESM.pdf]
