# Supplementary material for: Germline determinants of humoral immune response to HPV-16 protect against oropharyngeal cancer
Source: Nat Commun. 2021 Oct 12;12:5945. doi: 10.1038/s41467-021-26151-9 (PMC8511029; doi:10.1038/s41467-021-26151-9)
Supplement: Supplementary file 3 — Description of Additional Supplementary Files [file 41467_2021_26151_MOESM3_ESM.pdf]

Description of Additional Supplementary Files

File Name: Supplementary Data 1
Description: HPV(-) OPC regional meta-analysis results (P < 5 x10-6) from GWAS association statistics obtained from multivariate logistic regression assuming an additive genetic model with sex and principal components as covariates

File Name: Supplementary Data 2
Description: HPV(+) OPC regional meta-analysis results (P < 5 x10-6) from GWAS association statistics obtained from multivariate logistic regression assuming an additive genetic model with sex and principal components as covariates

File Name: Supplementary Data 3
Description: pooled HPV(-)OPC and OC results (P < 5 x10-6) from GWAS association statistics obtained from multivariate logistic regression assuming an additive genetic model with sex and principal components as covariates
