# Supplementary material for: Germline determinants of humoral immune response to HPV-16 protect against oropharyngeal cancer
Source: Nat Commun. 2021 Oct 12;12:5945. doi: 10.1038/s41467-021-26151-9 (PMC8511029; doi:10.1038/s41467-021-26151-9)
Supplement: Supplementary file 7 — Reporting Summary [file 41467_2021_26151_MOESM7_ESM.pdf]

nature portfolio

Corresponding author(s): Aida Ferreiro-Iglesias
Paul Brennan

Last updated by author(s): Aug 17, 2021

Reporting Summary

Nature Portfolio wishes to improve the reproducibility of the work that we publish. This form provides structure for consistency and transparency in reporting. For further information on Nature Portfolio policies, see our Editorial Policies and the Editorial Policy Checklist.

Statistics

For all statistical analyses, confirm that the following items are present in the figure legend, table legend, main text, or Methods section.

n/a	Confirmed	
☐	☒	The exact sample size (*n*) for each experimental group/condition, given as a discrete number and unit of measurement
☐	☒	A statement on whether measurements were taken from distinct samples or whether the same sample was measured repeatedly
☐	☒	The statistical test(s) used AND whether they are one- or two-sided<br>*Only common tests should be described solely by name; describe more complex techniques in the Methods section.*
☐	☒	A description of all covariates tested
☐	☒	A description of any assumptions or corrections, such as tests of normality and adjustment for multiple comparisons
☐	☒	A full description of the statistical parameters including central tendency (e.g. means) or other basic estimates (e.g. regression coefficient) AND variation (e.g. standard deviation) or associated estimates of uncertainty (e.g. confidence intervals)
☐	☒	For null hypothesis testing, the test statistic (e.g. *F*, *t*, *r*) with confidence intervals, effect sizes, degrees of freedom and *P* value noted<br>*Give P values as exact values whenever suitable.*
☒	☐	For Bayesian analysis, information on the choice of priors and Markov chain Monte Carlo settings
☒	☐	For hierarchical and complex designs, identification of the appropriate level for tests and full reporting of outcomes
☐	☒	Estimates of effect sizes (e.g. Cohen's *d*, Pearson's *r*), indicating how they were calculated

Our web collection on statistics for biologists contains articles on many of the points above.

Software and code

Policy information about availability of computer code

Data collection	methods/Subjects, specimens and genotypes (lines 400-423)
Data analysis	R software v.3.6.3, Haplo.stats R package (v.1.7.7)., UCSF Chimera v1.11. , SNP2HLA v1.0.3 package in Beagle software v3, PLINKv1.07, vmethods/Imputation and HLA fine mapping and association analyses and functional annotation

For manuscripts utilizing custom algorithms or software that are central to the research but not yet described in published literature, software must be made available to editors and reviewers. We strongly encourage code deposition in a community repository (e.g. GitHub). See the Nature Portfolio guidelines for submitting code & software for further information.

Data

Policy information about availability of data

All manuscripts must include a data availability statement. This statement should provide the following information, where applicable:
- Accession codes, unique identifiers, or web links for publicly available datasets
- A description of any restrictions on data availability
- For clinical datasets or third party data, please ensure that the statement adheres to our policy

Genotype data for the OPC and OC OncoArray study have been deposited at the database of Genotypes and Phenotypes (dbGaP) under accession phs001202.v1.p1 (https://www.ncbi.nlm.nih.gov/projects/gap/cgi-bin/study.cgi?study_id=phs001202.v1.p1) and it can be obtained by completing a proposal request form at dbGaP following instructions in https://dbgap.ncbi.nlm.nih.gov/aa/wga.cgi?page=login#l2 . The oral and pharyngeal GWAS summary statistics by cancer site and world region have been deposited in the IEU Open GWAS platform (https://gwas.mrcieu.ac.uk/) under the GWAs IDs: ieu-b-89, ieu-b-90, ieu-b-94, ieu-b-96, ieu-b-93, ieu-b-97, ieu-b-91, ieu-b-95 and ieu-b-98. Additional summary statistics data by HPV status generated in this study are provided in the Supplementary Information/ Dataset 1-3.

Protein Data Bank (RCSB PDB) structures for three-dimensional HLA ribbon models can be obtain under accession codes 3pdo (https://www.rcsb.org/structure/3PDO) and 2bpv (https://www.rcsb.org/structure/2BVP).
Genotype-Tissue Expression dataset (GTEx v8) is publicly available and can be downloaded following instructions at https://gtexportal.org/home/protectedDataAccess.

Field-specific reporting

Please select the one below that is the best fit for your research. If you are not sure, read the appropriate sections before making your selection.

- [x] Life sciences
- [ ] Behavioural & social sciences
- [ ] Ecological, evolutionary & environmental sciences

For a reference copy of the document with all sections, see nature.com/documents/nr-reporting-summary-flat.pdf

Life sciences study design

All studies must disclose on these points even when the disclosure is negative.

Sample size	We analyzed the largest and most extensive data of oral and oropharyngeal cancers with available genetic and HPV information that was collected through an international effort across continents to obtain the largest collection possible. In support of our sample size, these data has allow to detect several loci previously known and additional ones by using a rich collection of data, with convergent evidence of association from distinct approaches that identified the same variants with different approaches for the HPV(+) dataset and we have included a second dataset with potential genetic links with HPV(-) that allowed us to support the discovery of additional genome-wide significant SNPs
Data exclusions	Criteria for data inclusion in methods/Subjects, specimens and genotypes. All subjects not fitting this criteria were excluded.
Replication	An independent replication of some of our findings was recently published (discussion line 338) although no HPV information was available in this study. All attempts to replicate hese findings were successful to our knowledge
Randomization	Samples were allocated into cases and controls based on their diagnosis of cancer and their lack of medical history of this disease, respectively. Covariates were selected based on the epidemiology characteristics differing between the two groups and by performing a Principal Component Analyses (PCA) using their genetic data to control agnostically for unknown potential differences between the 2 groups.
Blinding	Investigators were blinded to the case–control status and cancer site of the subjects for genotype and plasma laboratory analyses and subsequent quality control checks.

Reporting for specific materials, systems and methods

We require information from authors about some types of materials, experimental systems and methods used in many studies. Here, indicate whether each material, system or method listed is relevant to your study. If you are not sure if a list item applies to your research, read the appropriate section before selecting a response.

Materials & experimental systems

n/a	Involved in the study	
☐	☒	Antibodies
☒	☐	Eukaryotic cell lines
☒	☐	Palaeontology and archaeology
☒	☐	Animals and other organisms
☐	☒	Human research participants
☒	☐	Clinical data
☒	☐	Dual use research of concern

Methods

n/a	Involved in the study	
☒	☐	ChIP-seq
☒	☐	Flow cytometry
☒	☐	MRI-based neuroimaging

Antibodies

Antibodies used	Samples were evaluated for antibodies against HPV proteins present naturally on the recruited samples.
Validation	methods/Serological analyses and HPV status determination (lines 441-458

Human research participants

Policy information about studies involving human research participants

Population characteristics	Relevant covariates for this study population are age at diagnosis/recruitment, sex, geographical region, smoking and drinking status and genotypic information
Recruitment	Samples were recruited and allocated into cases and controls based on their diagnosis of cancer and their lack of medical history of this disease, respectively. Both cases and controls were collected in a comparable number in each recruitment center and if controls were not available from an specific center, the additional set was of the same country of origin and ethnicity.
Ethics oversight	Each included study was approved by their corresponding local ethics committee/Institutional Review Board, and informed consent was obtained from all study participants. In addition, the International Agency for Research on Cancer Institutional Review Board (IARC-IRB; reference: 16-34) evaluated and approved inclusion of each contributing study into the current study

Note that full information on the approval of the study protocol must also be provided in the manuscript.
